# Effects of Fiber Orientation, Thermal Post-Curing, and Corrosive Environment on the Mechanical Properties of CFRP Laminates

**DOI:** 10.3390/polym18111270

**Published:** 2026-05-22

**Authors:** Štefan Kender, Janette Brezinová, Štefan Novotný, Petra Bejdová

**Affiliations:** 1 Department of Automotive Production, Faculty of Mechanical Engineering, Technical University of Košice, 04200 Kosice, Slovakia; stefan.kender@tuke.sk (Š.K.); stefan.novotny@tuke.sk (Š.N.); 2 Department of Medical and Clinical Biochemistry, Faculty of Medicine, Pavol Jozef Šafárik University in Košice, 04011 Kosice, Slovakia; bejda.peta@gmail.com

**Keywords:** carbon fiber-reinforced polymer, CFRP laminates, fiber orientation, mechanical properties, thermal post-curing, environmental degradation, tensile strength, impact toughness

## Abstract

Carbon fiber-reinforced polymer (CFRP) composites are widely used in engineering applications due to their high strength-to-weight ratio and corrosion resistance. However, their mechanical performance depends strongly on laminate architecture, processing conditions, and environmental exposure. This study investigates the effects of fiber orientation, thermal post-curing, and corrosive SO_2_ atmosphere on the mechanical properties of CFRP laminates. Three-layer carbon/epoxy laminates with 90°, 45°, and [90°/45°/90°] fiber orientations were manufactured by vacuum-assisted lamination. Selected specimens were post-cured at 80 °C for 10 h and exposed to sulfur dioxide according to ISO 3231. Tensile and Charpy impact tests showed that the 90° laminate exhibited the highest tensile strength (484 MPa), whereas the 45° laminate showed the lowest value due to shear-dominated load transfer. Post-curing increased tensile strength by approximately 10–30%, while exposure to the corrosive environment reduced both tensile strength and impact toughness. The observed behavior was associated with differences in load-transfer mechanism, possible increased degree of cure and/or residual stress relaxation after post-curing, and degradation of the epoxy–matrix and fiber–matrix interface after SO_2_ exposure. The results demonstrate that suitable selection of laminate architecture and thermal treatment can significantly improve the durability of CFRP structures intended for aggressive environments.

## 1. Introduction

Fiber-reinforced polymer (FRP) composites are widely used in modern engineering structures due to their excellent strength-to-weight ratio, corrosion resistance, and design flexibility. These materials have become increasingly important in aerospace, automotive, marine, and civil engineering applications where lightweight structures and high mechanical performance are required [1,2,3,4]. Among the different types of composite systems, carbon fiber-reinforced polymers (CFRPs) represent one of the most advanced material classes due to their superior stiffness, strength, and fatigue resistance compared to conventional structural materials [5,6,7].

Fiber-reinforced polymer composites may contain different types of reinforcing fibers and polymer matrices depending on the required structural performance. Glass fiber-reinforced polymers (GFRPs) are widely used because of their low cost and good corrosion resistance, although they generally provide lower stiffness and strength than carbon fiber composites. Aramid fiber-reinforced polymers (AFRPs) exhibit excellent impact resistance and damage tolerance but lower compressive stiffness. In comparison, CFRPs provide the highest specific stiffness and tensile strength, which makes them particularly suitable for lightweight load-bearing structures in aerospace and automotive applications. Similarly, the choice of polymer matrix significantly influences composite behavior. Polyester resins are economically attractive but exhibit lower mechanical properties and chemical resistance. Vinyl ester resins provide improved moisture and corrosion resistance, whereas epoxy matrices offer the best combination of mechanical strength, thermal stability, and fiber–matrix adhesion. For this reason, carbon fiber/epoxy systems are among the most commonly used composite materials in demanding engineering applications [8,9,10].

The mechanical performance of CFRP composites depends on several factors, including fiber type, matrix properties, fiber volume fraction, and the quality of the fiber–matrix interface [11,12]. However, one of the most important parameters affecting the mechanical behavior of laminated composites is the orientation of reinforcing fibers within the laminate structure [13,14,15]. Fiber orientation significantly influences the anisotropic mechanical response of composite materials and determines the ability of the laminate to transfer loads in different directions.

Previous studies have shown that unidirectional composites exhibit the highest stiffness and strength along the fiber direction, while off-axis fiber orientations can improve multidirectional load-bearing capacity and damage tolerance [16,17,18]. By adjusting the stacking sequence and fiber orientation angles, engineers can tailor the mechanical performance of composite laminates for specific structural applications [19,20]. Consequently, understanding the relationship between fiber orientation and mechanical properties is essential for optimizing composite design.

In laminated CFRP systems, the polymer matrix plays a crucial role by transferring loads between reinforcing fibers and protecting them from environmental influences. Epoxy resins are commonly used as matrix materials due to their good mechanical properties, chemical resistance, and strong adhesion to carbon fibers [21,22]. Nevertheless, the mechanical performance of epoxy-based composites can be affected by manufacturing parameters such as curing temperature, curing time, and post-curing heat treatment [23]. Heat treatment may increase the degree of cross-linking within the epoxy matrix, thereby improving stiffness and strength while reducing residual stresses generated during the curing process [24,25].

Environmental conditions represent another important factor affecting the long-term durability of polymer composites. During service, CFRP components may be exposed to humidity, temperature variations, and chemically aggressive environments, which can lead to degradation of the polymer matrix and weakening of the fiber–matrix interface [26,27]. Moisture diffusion into epoxy matrices can cause plasticization, swelling, and microcracking, ultimately reducing the mechanical performance of the composite material [28,29,30].

Several studies have investigated the influence of environmental exposure on the mechanical properties of CFRP composites. For example, moisture absorption has been reported to decrease tensile and flexural properties due to matrix softening and interfacial degradation [31,32]. Similarly, corrosive environments and elevated temperatures can accelerate aging processes in epoxy matrices and significantly affect the mechanical behavior of composite structures [33].

Environmental aging of polymer composites is generally controlled by the coupled action of temperature, moisture, and chemically aggressive media. Elevated temperature accelerates the diffusion of water molecules into the polymer matrix and promotes degradation processes at the fiber–matrix interface. Moisture uptake in epoxy-based composites usually follows Fickian or near-Fickian diffusion behavior and depends on matrix chemistry, fiber architecture, and the presence of pores and microcracks. Absorbed water causes plasticization and swelling of the epoxy matrix, reduces the glass transition temperature, and may induce local microcracking and interfacial debonding between fibers and matrix. In corrosive environments containing acidic or alkaline species, these effects are further intensified because aggressive ions penetrate through the damaged matrix and accelerate the deterioration of the interfacial region. As a result, the long-term mechanical performance of composite laminates gradually decreases, particularly in terms of tensile strength, stiffness, and interlaminar properties. Similar mechanisms of moisture diffusion and environmentally induced degradation have been reported in recent studies dealing with long-term durability of fiber-reinforced polymer composites [34,35,36,37].

Despite extensive research on composite materials, the combined effects of fiber orientation, thermal treatment, and environmental exposure on the mechanical properties of CFRP laminates are still not fully understood. In particular, laminate architectures with different fiber orientations may exhibit different degradation mechanisms due to variations in internal stress distribution and moisture diffusion pathways within the composite structure.

Therefore, further experimental investigations are required to better understand how laminate configuration influences the mechanical behavior of CFRP composites subjected to environmental degradation. Such knowledge is essential for improving the reliability and durability of composite structures used in demanding engineering applications.

The present study focuses on the evaluation of selected mechanical properties of carbon fiber-reinforced polymer composites with different fiber orientations. Laminated specimens consisting of three layers of carbon fiber reinforcement and an epoxy matrix were prepared with various fiber orientations (90°, 45°, and 90°/45°/90°). In addition, the influence of thermal post-treatment and exposure to a corrosive environment was investigated. The main objective of this research is to experimentally determine how fiber orientation and environmental conditions affect the mechanical properties of CFRP laminates.

Carbon fiber-reinforced epoxy laminates were selected in the present study because this material system provides a favorable combination of high specific strength, stiffness, corrosion resistance, and strong interfacial bonding between carbon fibers and the epoxy matrix. These characteristics make CFRP/epoxy composites particularly suitable for structural applications in the aerospace, automotive, and transportation industries, where components may operate under aggressive environmental conditions. The novelty of the present work lies in the combined evaluation of three factors that are usually studied separately, namely fiber orientation, thermal post-curing, and exposure to a humid SO_2_-containing environment. Three laminate architectures (90°, 45°, and 90°/45°/90°) were systematically compared, and both tensile strength and impact toughness were evaluated in order to provide a more comprehensive understanding of the mechanical performance and environmental durability of CFRP laminates.

## 2. Materials and Methods

### 2.1. Used Materials

Carbon fiber-reinforced polymer (CFRP) laminates were prepared using carbon fiber fabric with an aerial weight of 160 g·m^−2^ supplied by (Havel Composites CZ s.r.o., Svésedlice, Czech Republic). The polymer matrix consisted of epoxy resin L285 with hardener H286 (Havel Composites CZ s.r.o., Svésedlice, Czech Republic), which is commonly used for structural composite applications due to its good mechanical properties and chemical resistance.

Auxiliary materials used during the manufacturing process included a peel ply (Interglas 98685, 93 g·m^−2^) and a breather fabric (200 g·m^−2^) to ensure proper resin distribution and removal of excess resin during vacuum processing. A glass plate coated with release wax (FRP HL-603) (Havel Composites CZ s.r.o., Svésedlice, Czech Republic) was used as the base surface for laminate fabrication.

The basic properties of the raw materials used for laminate preparation are summarized in Table 1.

### 2.2. Preparation of Composite Laminates

**Figure 1:**
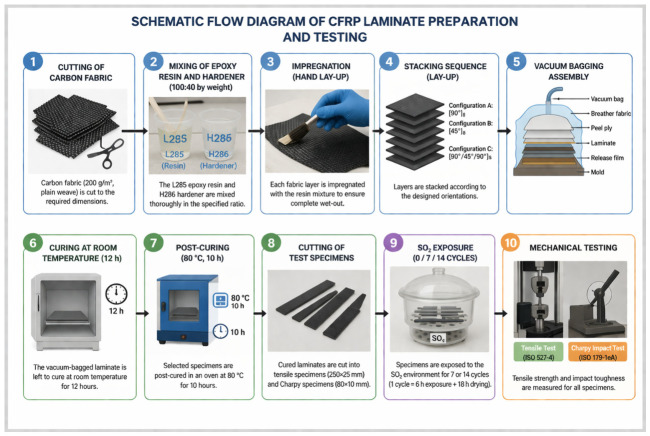
Experimental procedure used for fabrication of CFRP laminates with different fiber orientations, subsequent thermal treatment, SO_2_ exposure, and mechanical testing.

The preparation procedure of the CFRP laminates is schematically illustrated in Figure 1.

Composite laminates were manufactured from three layers of carbon fiber fabric impregnated with epoxy resin. The aim of the study was to evaluate the influence of fiber orientation on the mechanical properties of the composite material; therefore, three laminate configurations were prepared:

- 90° configuration: all three layers oriented at 90° relative to the specimen axis;
- 45° configuration: all three layers oriented at 45°;
- 90°/45°/90° configuration: outer layers oriented at 90° and middle layer at 45°.

The resin system was mixed according to the manufacturer’s recommendation at a ratio of 100 parts resin to 40 parts hardener by weight. For one production batch, approximately 110 g of epoxy resin and 45 g of hardener were used.

The resin was applied to the carbon fiber fabric using a foam roller to achieve uniform impregnation. After the impregnation of each layer, the subsequent layer was placed and impregnated in the same manner. After stacking the three layers, trapped air bubbles were removed and excess resin was absorbed using the auxiliary fabrics.

The laminate stack was covered with peel ply and breather fabric, sealed with a vacuum foil, and connected to a vacuum pump. Vacuum pressure ensured consolidation of the laminate and removal of excess resin. The laminates were cured under vacuum at room temperature for 12 h.

The average thickness of the fabricated three-layer laminates was approximately 0.75 ± 0.05 mm. Based on the areal weight and density of the carbon fabric together with the measured laminate mass, the fiber volume fraction of the CFRP laminates was estimated to be in the range of 50–55 vol.%.

After curing, the composite plates were removed from the mold and cut into test specimens using precision shears according to the required specimen dimensions.

### 2.3. Thermal Post-Curing

To investigate the influence of additional heat treatment, selected specimens were subjected to thermal post-curing. According to the technical documentation of the epoxy system L285/H286, improved thermal and mechanical properties can be achieved by post-curing at elevated temperatures.

Post-curing was performed in an electric laboratory oven (Ecocell) (MMM Medcenter Einrichtungen GmbH, Munich, Germany) at 80 °C for 10 h. The specimens were placed on a metal grid inside the preheated furnace to ensure uniform heat distribution. After completion of the heat treatment, the specimens were removed from the furnace and allowed to cool naturally at room temperature.

Although post-curing is often recommended for epoxy-based composites, it is not always applied in practical manufacturing due to increased processing time and energy consumption. Therefore, the present study compares laminates cured only at room temperature with laminates additionally post-cured at 80 °C in order to quantify the contribution of this additional technological step to the mechanical performance and environmental durability of CFRP laminates.

### 2.4. Exposure to Corrosive Environment

The SO_2_-containing environment was selected to simulate industrial and urban atmospheres with elevated concentrations of acidic pollutants originating from combustion processes, exhaust gases, and industrial emissions. Such environments are relevant for CFRP structures used in transportation, civil engineering, and industrial applications, where long-term exposure to humidity and sulfur-containing pollutants may accelerate degradation of the polymer–matrix and fiber–matrix interface. To evaluate the environmental durability of the composite material, the specimens were exposed to a humid atmosphere containing sulfur dioxide (SO_2_) according to ISO 3231 standard [38].

The exposure tests were carried out in a condensation chamber KK 260 (Kambič Laboratory Equipment, Semič, Slovenia),at a temperature of 40 ± 3 °C and relative humidity of 100%, with a sulfur dioxide concentration of 2 mg·L^−1^. The specimens were suspended on acrylic rods to maintain the required spacing between samples.

Two exposure durations were investigated:

- 7 cycles (168 h),
- 14 cycles (336 h).

One cycle corresponded to 24 h of exposure in the corrosive environment. Seven and fourteen exposure cycles were selected to simulate intermediate and long-term environmental degradation, enabling evaluation of the progressive changes in mechanical properties of the CFRP laminates during accelerated SO_2_ aging.

### 2.5. Used Experimental Methods

Tensile Testing

Tensile tests were performed to determine the tensile properties of the CFRP laminates according to EN ISO 527-4 standard [39]. The tests were conducted using a universal testing machine TIRA Test 2300 (VEB TIW Rauenstein, Rauenstein, Germany) equipped with grips capable of transmitting loads up to 10 kN.

The crosshead speed during the test was 10 mm·min^−1^. Tensile strength was determined from the maximum load recorded during the test.

Impact Testing

Impact resistance of the composites was evaluated using the Charpy impact test according to EN ISO 179-1 standard [40]. The tests were performed using a PSW 60/500 (PSW Prüf- und Schwingtechnik GmbH, Elsteraue, Germany) Charpy pendulum impact tester.

The impact toughness was determined from the difference between the potential energies of the pendulum before and after fracture of the specimen. The absorbed energy was read directly from the calibrated scale of the testing device.

For each laminate configuration, specimens were divided into different experimental groups to evaluate the effects of heat treatment and environmental exposure. Each group contained five specimens, resulting in a total of 180 test specimens. Although five specimens per group were considered sufficient to identify the main trends in mechanical behavior, a larger sample size would provide a more robust statistical evaluation of the observed differences. The experimental design included the following variables:

- fiber orientation (90°, 45°, 90°/45°/90°),
- thermal post-curing (with or without heat treatment),
- exposure to corrosive environment (0, 7, and 14 cycles).

This experimental setup allowed systematic evaluation of the combined effects of laminate architecture, thermal processing, and environmental degradation on the mechanical properties of CFRP composites.

Statistical analysis of the experimental results was performed using one-way ANOVA with significance level *p* < 0.05.

## 3. Results

### 3.1. Tensile Strength of CFRP Laminates

**Figure 2:**
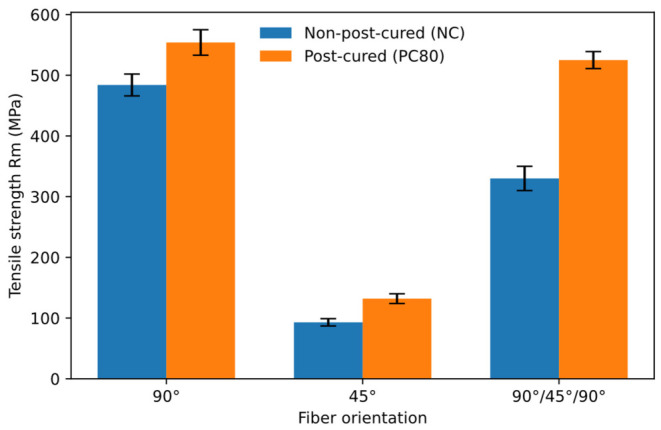
Effect of fiber orientation and post-curing (80 °C, 10 h) on the tensile strength of CFRP laminates.

The tensile strength of the investigated CFRP laminates strongly depended on the orientation of reinforcing fibers and applied curing regime. Effect of fiber orientation and post-curing on tensile strength is shown in Figure 2. In reference condition without post-curing, highest tensile strength was observed for orthogonal laminate configuration 90°, reaching 484 MPa. This behavior is attributed to alignment of carbon fibers parallel to loading direction, allowing fibers to carry majority of applied tensile loads. In contrast, laminate with fibers oriented at 45° exhibited lowest tensile strength (93 MPa), since applied load was transferred predominantly through in-plane shear stresses acting in epoxy matrix and at fiber–matrix interface. Hybrid laminate configuration [90°/45°/90°] showed intermediate behavior with tensile strength of 330 MPa, reflecting combined contribution of fiber-dominated and matrix-dominated load-transfer mechanisms. After post-curing at 80 °C for 10 h, significant increase in tensile strength was observed for all laminate architectures. Tensile strength of 90° laminate increased to 554 MPa, representing an increase of approximately 14% compared with reference condition. Similarly, tensile strength of 45° laminate increased from 93 MPa to 132 MPa, corresponding to an increase of approximately 42%. Most pronounced improvement was observed for hybrid configuration [90°/45°/90°], where tensile strength increased from 330 MPa to 525 MPa, representing an increase of approximately 59%.

#### 3.1.1. Tensile Strength After Corrosion Exposure

**Figure 3:**
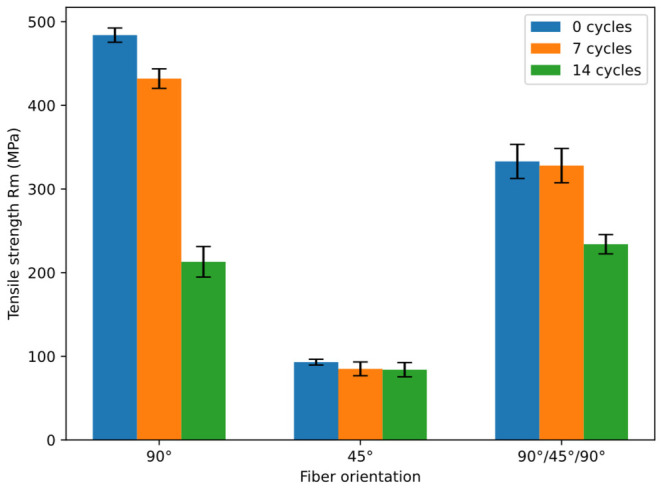
Effect of corrosion exposure on tensile strength of CFRP laminates with different fiber orientations. Specimens were exposed in a corrosion chamber containing SO_2_ for 7 and 14 cycles, according to ISO 3231.

Influence of corrosive environment on tensile strength of CFRP laminates is presented in Figure 3. Specimens were exposed in a corrosion chamber containing SO_2_ for 7 and 14 cycles in order to simulate aggressive atmospheric conditions. Results indicate that corrosion exposure led to progressive decrease in tensile strength for all laminate configurations. Most pronounced reduction was observed for 90° laminates, where tensile strength decreased from 484 MPa in reference condition to 432 MPa after 7 cycles and further to 213 MPa after 14 cycles, corresponding to overall reduction of approximately 56%.

Hybrid laminate configuration [90°/45°/90°] also exhibited significant decrease in tensile strength after corrosion exposure. Average tensile strength decreased from 333 MPa in reference condition to 328 MPa after 7 cycles and further to 234 MPa after 14 cycles, representing reduction of approximately 30%. Laminate with fibers oriented at 45° showed the lowest tensile strength in reference condition (93 MPa). After corrosion exposure, tensile strength slightly decreased to 85 MPa after 7 cycles and 84 MPa after 14 cycles, corresponding to reduction of approximately 10%. Observed decrease in tensile strength can be attributed to degradation of epoxy matrix caused by penetration of moisture and corrosive species into composite structure. Manufacturing defects such as pores and microcracks likely facilitated transport of corrosive medium into laminate interior, resulting in weakening of the fiber–matrix interface and reduced load-transfer efficiency within the laminate.

#### 3.1.2. Tensile Strength of Post-Cured Laminates After Corrosion Exposure

**Figure 4:**
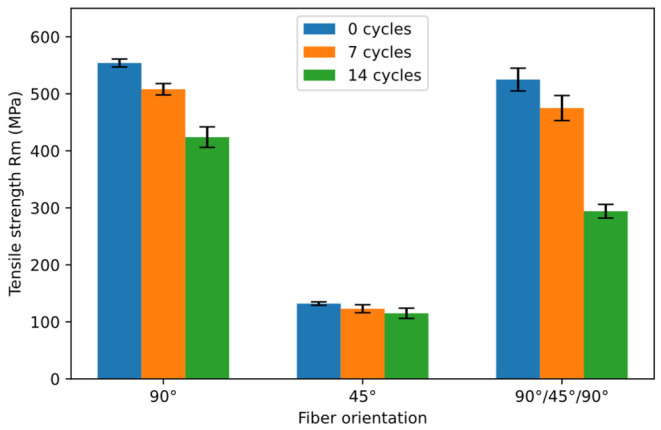
Influence of corrosion exposure on tensile strength Rm of post-cured CFRP laminates with different fiber orientations (90°, 45°, and 90°/45°/90°). Specimens were post-cured at 80 °C and subsequently exposed in a corrosion chamber containing SO_2_ for 7 and 14 cycles according to ISO 3231.

Influence of corrosive environment on tensile strength of post-cured CFRP laminates is shown in Figure 4. Similar trend as in non-post-cured specimens was observed, where increasing exposure time resulted in progressive decrease in tensile strength. Highest tensile strength in reference condition was recorded for the 90° laminate, reaching approximately 554 MPa. After exposure in the corrosive environment, tensile strength slightly decreased to 508 MPa after 7 cycles and further to 424 MPa after 14 cycles, corresponding to reduction of approximately 24%. Laminate with fibers oriented at 45° exhibited significantly lower tensile strength in reference condition (132 MPa). After corrosion exposure, tensile strength decreased to 123 MPa after 7 cycles and 115 MPa after 14 cycles, corresponding to reduction of approximately 13%. Hybrid laminate configuration [90°/45°/90°] showed intermediate behavior with tensile strength of 525 MPa in reference condition. After corrosion exposure, tensile strength decreased to 475 MPa after 7 cycles and 294 MPa after 14 cycles, representing reduction of approximately 44%. Decrease in tensile strength after corrosion exposure can be attributed to degradation of epoxy matrix and weakening of fiber–matrix interface caused by penetration of moisture and corrosive species into composite structure.

### 3.2. Impact Toughness of CFRP Laminates

**Figure 5:**
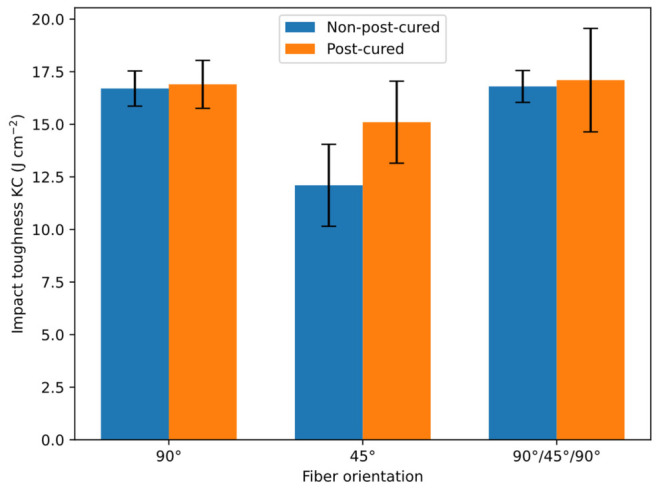
Influence of fiber orientation and post-curing on impact toughness KC of CFRP laminates measured by Charpy impact test.

Impact toughness of investigated CFRP laminates was evaluated using Charpy impact testing in order to assess resistance of composite structures to dynamic loading. Results are presented in Figure 5. Impact toughness strongly depends on laminate architecture and fiber orientation. Hybrid laminate configuration [90°/45°/90°] exhibited highest impact toughness in reference condition, reaching approximately 16.8 J·cm^−2^, followed by [90°]_3_ laminate with 16.7 J·cm^−2^. Laminate with fibers oriented at 45° showed significantly lower impact toughness of approximately 12.1 J·cm^−2^, indicating increased susceptibility to matrix-dominated fracture mechanisms. Post-curing at 80 °C for 10 h resulted in moderate increase in impact toughness for all laminate configurations. Impact toughness of 90° laminate increased slightly to 16.9 J·cm^−2^, while 45° laminate exhibited more pronounced increase to 15.1 J·cm^−2^, corresponding to improvement of approximately 25%. Hybrid laminate [90°/45°/90°] reached 17.1 J·cm^−2^ after post-curing. Observed improvement in impact resistance can be attributed to increased cross-link density of epoxy matrix after thermal treatment, which enhances energy absorption capacity and improves load transfer between reinforcement layers during impact loading.

#### 3.2.1. Impact Toughness After Corrosion Exposure (Non-Post-Cured Laminates)

**Figure 6:**
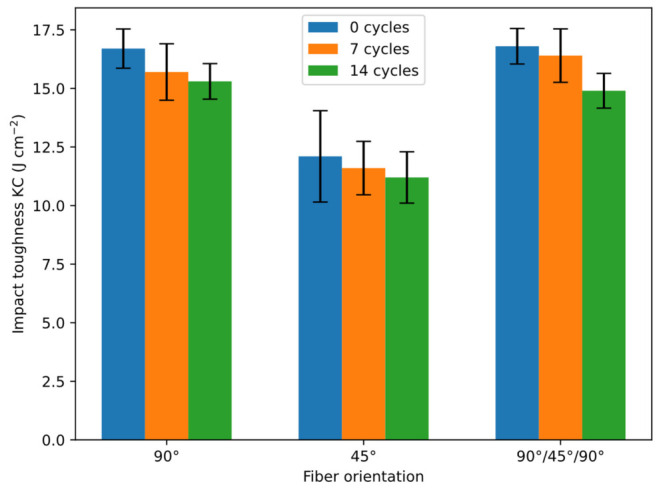
Influence of corrosion exposure on impact toughness KC of CFRP laminates with different fiber orientations. Specimens were exposed in a corrosion chamber containing SO_2_ for 7 and 14 cycles according to ISO 3231.

Impact toughness of CFRP laminates after exposure to corrosive environment was evaluated in order to determine influence of environmental degradation on dynamic mechanical properties. Results for non-post-cured specimens are presented in Figure 6. Results indicate that exposure to corrosive environment resulted in gradual decrease in impact toughness with increasing number of exposure cycles. In reference condition, laminate with fiber orientation 90° exhibited impact toughness of approximately 16.7 J·cm^−2^. After exposure in corrosive environment, impact toughness decreased to 15.7 J·cm^−2^ after 7 cycles and further to 15.3 J·cm^−2^ after 14 cycles. Similar trend was observed for laminate configuration 90°/45°/90°, where impact toughness decreased from 16.8 J·cm^−2^ in reference condition to 16.4 J·cm^−2^ after 7 cycles and 14.9 J·cm^−2^ after 14 cycles. Laminate with fibers oriented at 45° exhibited lower impact toughness compared with other laminate architectures. In reference condition, impact toughness reached approximately 12.1 J·cm^−2^, which decreased to 11.6 J·cm^−2^ after 7 cycles and 11.2 J·cm^−2^ after 14 cycles. Observed decrease in impact toughness can be attributed to degradation of epoxy matrix and weakening of fiber–matrix interface caused by penetration of moisture and corrosive species into composite structure during corrosion exposure.

#### 3.2.2. Impact Toughness After Corrosion Exposure (Post-Cured Laminates)

**Figure 7:**
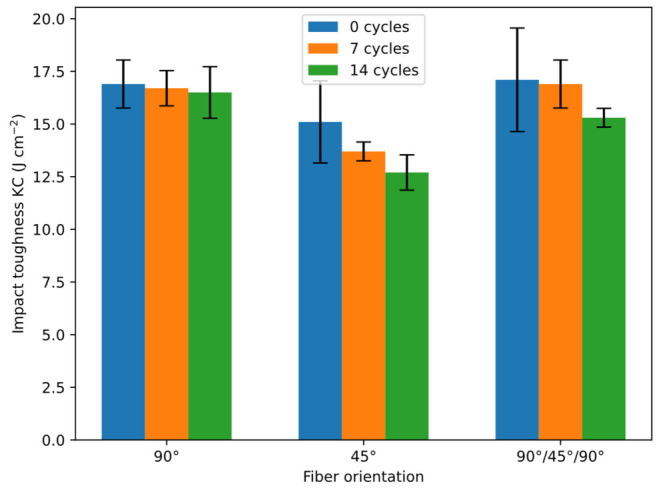
Influence of corrosion exposure on impact toughness KC of post-cured CFRP laminates with different fiber orientations (90°, 45°, and 90°/45°/90°). Specimens were post-cured at 80 °C and subsequently exposed in a corrosion chamber containing SO_2_ for 7 and 14 cycles according to ISO 3231.

Influence of corrosive environment on impact toughness of post-cured CFRP laminates is presented in Figure 7. Similar trend for non-post-cured specimens was observed, where increasing number of corrosion cycles resulted in gradual decrease in impact toughness. For 90° laminate, impact toughness decreased slightly from 16.9 J·cm^−2^ in reference condition to 16.7 J·cm^−2^ after 7 cycles and 16.5 J·cm^−2^ after 14 cycles. More pronounced reduction was observed for laminate with 45° fiber orientation, where impact toughness decreased from 15.1 J·cm^−2^ in reference condition to 13.7 J·cm^−2^ after 7 cycles and further to 12.7 J·cm^−2^ after 14 cycles. Hybrid laminate configuration [90°/45°/90°] exhibited highest impact toughness in reference condition (17.1 J·cm^−2^), which decreased to 16.9 J·cm^−2^ after 7 cycles and 15.3 J·cm^−2^ after 14 cycles. Observed decrease in impact toughness can be attributed to degradation of epoxy matrix and weakening of fiber–matrix interface caused by penetration of moisture and corrosive species into composite structure.

### 3.3. Failure Analysis of CFRP Specimens After Tensile Testing

**Figure 8:**
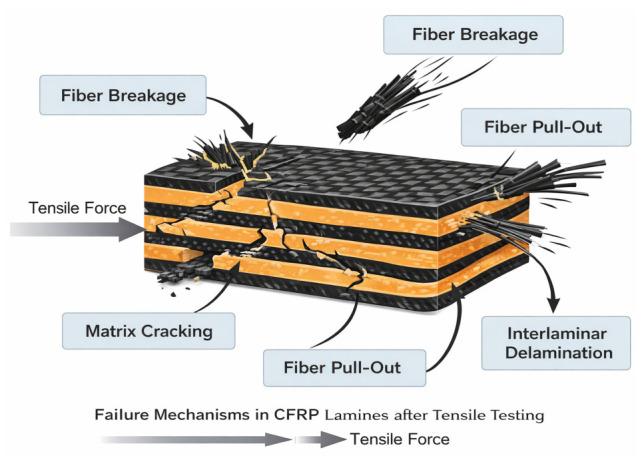
Schematic illustration of typical tensile failure mechanisms in CFRP laminates.

**Figure 9:**
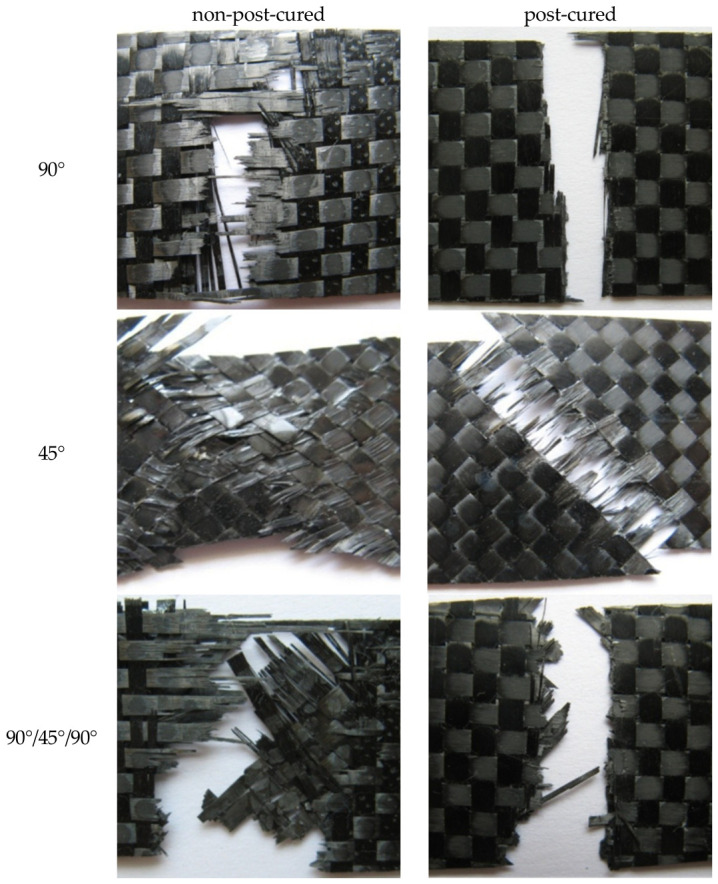
Typical failure modes of CFRP laminates after tensile testing for different fiber orientations and curing conditions. Observed fracture mechanisms include fiber breakage, matrix cracking, fiber pull-out, and interlaminar delamination.

To facilitate interpretation of fracture surfaces, schematic representation of principal tensile failure mechanisms observed in CFRP laminates is shown in Figure 8. Representative fracture morphologies of tested specimens are subsequently presented in Figure 9. Fracture characteristics strongly depended on fiber orientation and applied post-curing treatment.

**Figure 10:**
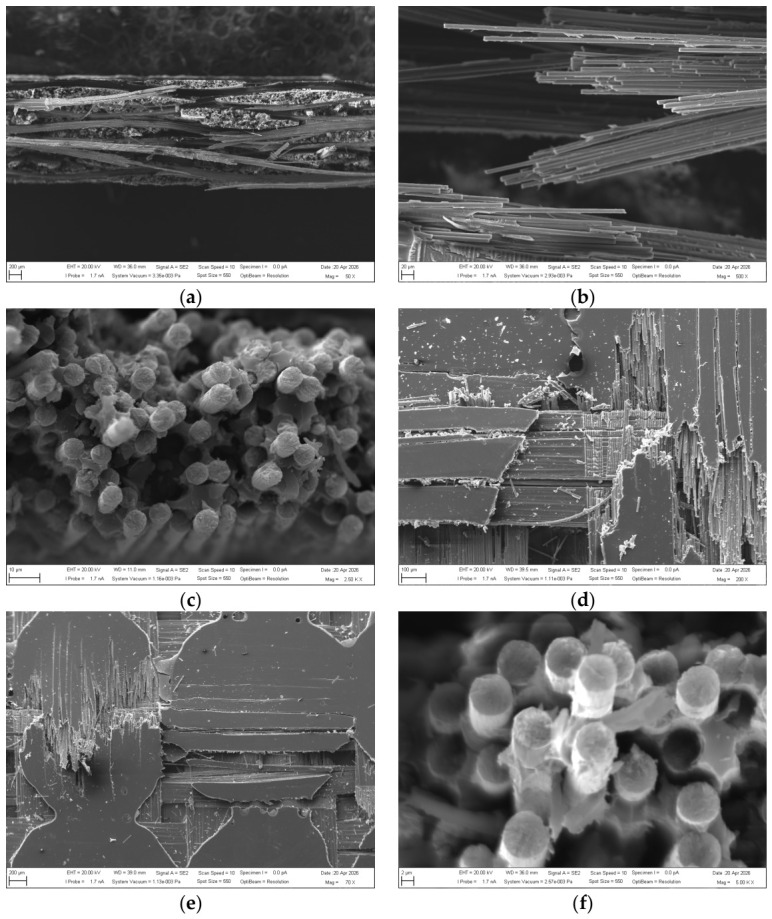
SEM fractography of CFRP laminates after mechanical testing: (**a**) Overall morphology of the fracture surface in the reference specimen; (**b**) broken and partially pulled-out carbon fibers; (**c**) post-cured specimen showing a more compact fracture morphology and shorter fiber pull-out lengths; (**d**) specimen exposed to SO_2_ showing more pronounced interfacial degradation and matrix cracking; (**e**) local fiber/matrix debonding and cavity formation after fiber pull-out; (**f**) high-magnification detail of brittle fracture of a carbon fiber.

SEM fractography of representative fracture surfaces is shown in Figure 10. The SEM images provide more detailed evidence of the failure mechanisms responsible for the differences in tensile strength between the laminates investigated. SEM analysis of the fracture surfaces revealed that the failure mechanism of the CFRP laminates was governed by a combination of carbon fiber fracture, fiber pull-out, and local debonding at the fiber/matrix interface. Figure 10a shows the overall morphology of the fractured region, where the crack propagated predominantly along the fiber direction. As shown in Figure 10b, numerous carbon fibers remained partially pulled out from the epoxy matrix. The different pull-out lengths indicate progressive failure of the reinforcement and suggest that crack propagation occurred through repeated interfacial debonding followed by fiber fracture.

Compared with the reference laminate, the post-cured specimens exhibited shorter fiber pull-out lengths and a more compact fracture morphology (Figure 10c), suggesting improved interaction between the carbon fibers and the epoxy matrix. In contrast, the specimens exposed to the SO_2_ environment showed more pronounced interfacial separation, cavities after fiber pull-out, and local matrix cracking (Figure 10d), which suggests progressive degradation of the fiber/matrix interface during environmental cycling. Figure 10e provides a detailed view of the interfacial region after failure. Local cavities remaining after fiber pull-out and partial separation between the carbon fibers and the surrounding epoxy matrix are clearly visible. These features confirm that interfacial debonding contributed significantly to crack propagation and subsequent loss of load-bearing capacity. A high-magnification image of the fractured fiber ends is shown in Figure 10f. The relatively flat and smooth fracture surfaces without visible plastic deformation indicate predominantly brittle fracture of the carbon fibers.

For laminates with fibers oriented at 90°, fracture was primarily characterized by fiber breakage accompanied by limited fiber pull-out. This failure mode indicates efficient stress transfer between carbon fibers and epoxy matrix. Post-cured specimens exhibited more compact fracture surfaces with reduced fiber pull-out, which is consistent with the SEM observations shown in Figure 10. In laminates with 45° fiber orientation, failure occurred predominantly through matrix cracking and shear-dominated fracture mechanisms. Crack propagation followed fiber directions, resulting in progressive interlaminar delamination and extensive fiber sliding within the matrix. Such fracture behavior is consistent with lower tensile strength observed for this laminate configuration. Hybrid laminates [90°/45°/90°] exhibited a more complex failure pattern involving fiber breakage, matrix cracking, interfacial debonding, and local delamination between adjacent layers. Interaction between differently oriented reinforcement layers resulted in multiple crack-propagation paths and gradual structural failure of the laminate. Overall, post-curing resulted in more compact fracture morphology and reduced matrix fragmentation, indicating improved structural integrity of the composite system. Similar fracture mechanisms in carbon fiber-reinforced polymer laminates under tensile loading have been widely reported in previous studies, where failure processes are typically governed by a combination of fiber fracture, matrix cracking, interfacial debonding, and delamination between reinforcement layers [37,41,42,43].

### 3.4. Failure Analysis of CFRP Specimens After Impact Toughness

**Figure 11:**
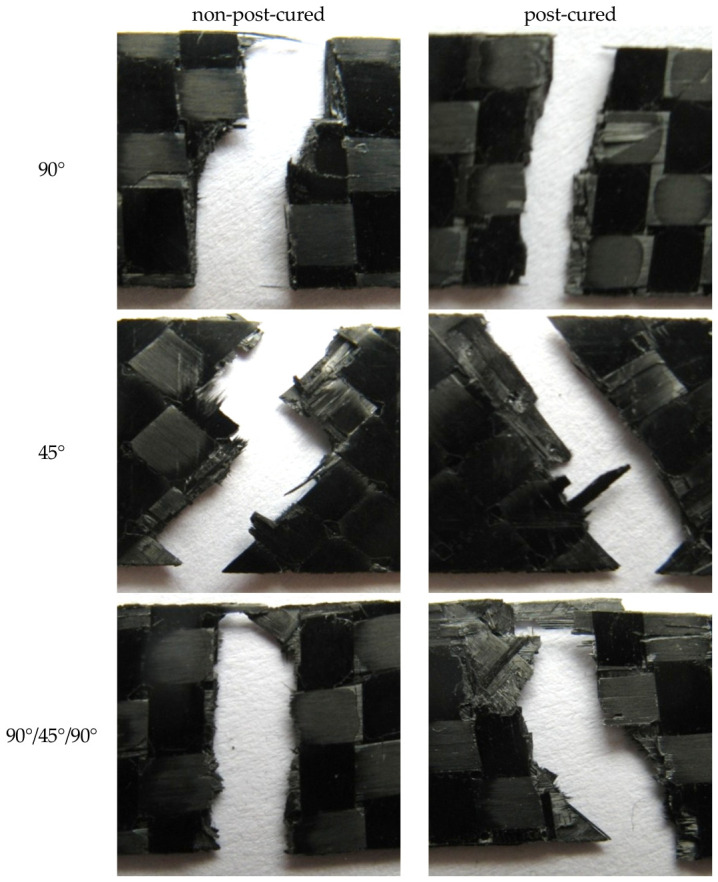
Representative fracture morphologies of CFRP laminates after Charpy impact testing for different fiber orientations and curing conditions.

Representative fracture morphologies of CFRP laminates after Charpy impact testing are presented in Figure 11. Impact loading produced more complex and heterogeneous fracture surfaces compared with tensile failure due to the combined action of bending, shear stresses, and dynamic crack propagation within the laminate structure. For laminates with fibers oriented at 90°, fracture surfaces were primarily characterized by localized fiber breakage accompanied by limited matrix cracking. The relatively straight fracture paths indicate that the majority of the impact energy was absorbed by fiber fracture mechanisms. Post-cured specimens exhibited slightly more compact fracture surfaces, suggesting improved fiber–matrix interfacial bonding which may be associated with improved matrix integrity and fiber–matrix interaction after thermal treatment. In laminates with 45° fiber orientation, failure was dominated by matrix cracking and extensive fiber sliding within the polymer matrix. The oblique orientation of reinforcing fibers promoted shear-dominated deformation during impact loading, which resulted in progressive crack propagation along the fiber directions and partial delamination between reinforcement layers. This fracture mechanism corresponds well with the lower impact toughness values observed for this laminate configuration. Hybrid laminates with stacking sequence [90°/45°/90°] exhibited the most complex fracture morphology. Damage mechanisms included a combination of fiber breakage, matrix cracking, interfacial debonding, and interlaminar delamination. Interaction between layers with different fiber orientations created multiple crack propagation paths and promoted gradual energy dissipation during fracture. As a result, hybrid laminates demonstrated the highest impact toughness among the investigated laminate architectures. Comparison of post-cured and non-post-cured specimens revealed that thermal post-curing slightly improved the structural integrity of the fracture surfaces, which may be associated with an increased degree of cure and improved stress transfer between the fibers and the matrix. However, the overall fracture mechanisms remained similar for both curing conditions. Overall, the observed fracture characteristics confirm that laminate architecture plays a key role in determining the impact damage mechanisms of CFRP composites. Laminates containing differently oriented reinforcement layers are capable of distributing stresses more effectively during dynamic loading, which contributes to improved energy absorption and higher impact resistance of the composite structure.

## 4. Discussion

The results of the present study demonstrate that fiber orientation, thermal post-curing, and environmental exposure significantly influence the mechanical performance of CFRP laminates. Among the investigated parameters, fiber orientation proved to be the most important factor affecting the tensile behavior of the composite material.

The highest tensile strength was observed for laminates with fibers oriented at 90° relative to the loading direction. This behavior is consistent with the fundamental mechanics of fiber-reinforced composites, where reinforcing fibers carry the majority of the applied load when aligned with the loading direction. Similar findings have been reported in several studies investigating anisotropic mechanical properties of laminated composites, where tensile strength along the fiber direction significantly exceeds off-axis orientations due to efficient load transfer between fibers and matrix [13,14,15].

In contrast, laminates with fibers oriented at 45° exhibited substantially lower tensile strength. In such configurations, the applied tensile load is primarily transferred through shear stresses in the polymer matrix and at the fiber–matrix interface. As a result, the mechanical response becomes matrix-dominated, which explains the reduced strength observed in the present experiments. Similar reductions in tensile performance of off-axis laminates have been reported by Zhang et al. [43], who demonstrated that shear-dominated failure mechanisms significantly reduce the load-bearing capacity of carbon fiber-reinforced laminates.

Thermal post-curing was found to significantly improve the tensile properties of the investigated CFRP laminates. The increase in tensile strength observed after post-curing may be associated with an increased degree of cross-linking in the epoxy matrix and/or relaxation of residual stresses generated during room-temperature curing. Similar improvements in mechanical performance after post-curing have been reported in previous studies on epoxy-based composites, where additional heat treatment improved tensile strength and interfacial bonding between fiber and matrix [44]. Additional thermal treatment may reduce the amount of unreacted groups in the polymer network, decrease chain mobility, and improve the adhesion between carbon fibers and the surrounding matrix. As a result, stress transfer becomes more efficient and failure mechanisms, such as fiber pull-out and interfacial debonding, are reduced. This effect was particularly pronounced for the hybrid laminate configuration, where improved bonding between differently oriented layers enhanced the overall load-bearing capacity of the laminate. The effect of fiber orientation on the mechanical response is associated with different load-transfer mechanisms within the laminate. In the 90° configuration, the applied tensile load is transferred mainly through the carbon fibers, which explains the highest tensile strength. In contrast, the 45° configuration is dominated by matrix shear stresses and interfacial stresses, resulting in lower strength and greater sensitivity to matrix cracking. The hybrid [90°/45°/90°] laminate combines both mechanisms and provides improved energy absorption during impact loading because cracks propagating through one layer are partially arrested or deflected by adjacent layers with different fiber orientation.

Environmental exposure in a humid SO_2_ atmosphere resulted in a gradual decrease in both tensile strength and impact toughness. This degradation can be attributed to the penetration of moisture and corrosive species into the composite structure. Moisture diffusion into epoxy matrices may lead to plasticization, swelling, and microcrack formation, which weaken the fiber–matrix interface and reduce the mechanical integrity of the composite laminate. Similar degradation mechanisms have been widely reported for polymer matrix composites exposed to aggressive environmental conditions [26,45,46]. Exposure to the humid SO_2_ environment accelerated degradation of the epoxy–matrix and fiber–matrix interface. Water molecules and corrosive species penetrated into the laminate through pores, microcracks, and interlaminar regions, causing matrix plasticization, swelling, and local debonding. These processes gradually reduced the ability of the composite to transfer stresses between adjacent fibers and layers, which explains the observed decrease in tensile strength and impact toughness after prolonged exposure.

The observed decrease in tensile strength after prolonged SO_2_ exposure may result from a combination of reversible matrix plasticization caused by moisture uptake and irreversible chemical degradation of the epoxy matrix and fiber–matrix interphase. Previous studies have shown that moisture absorption in epoxy systems may initially lead to reversible plasticization and reduction in the glass transition temperature, whereas prolonged exposure may also induce irreversible chemical changes and chain scission within the polymer network [34]. However, the present study does not allow these mechanisms to be distinguished unambiguously. The influence of corrosion exposure was particularly pronounced for laminates with fibers oriented at 90°, where a significant decrease in tensile strength was observed after prolonged environmental exposure. This behavior may be associated with the higher load-transfer efficiency along the fiber direction, making any degradation of the matrix or interface more critical for overall structural performance. In contrast, laminates with 45° orientation already exhibited matrix-dominated mechanical behavior, which may explain the smaller relative decrease in tensile strength after environmental exposure.

Impact toughness results revealed a slightly different trend compared to tensile properties. While fiber orientation remained an important parameter, the influence of post-curing on impact toughness was less pronounced than in the case of tensile strength. Impact loading involves complex fracture processes including matrix cracking, fiber pull-out, and delamination between adjacent layers. The hybrid laminate configuration [90°/45°/90°] showed the highest impact toughness, which can be explained by the interaction of differently oriented reinforcement layers that promote multiple crack propagation paths and higher energy absorption during fracture.

The different effect of post-curing on impact toughness for the 45° and 90° laminates may be related to their distinct fracture mechanisms. In the 90° configuration, impact failure was dominated mainly by fiber breakage; therefore, changes in matrix properties after post-curing only had a limited effect on the overall impact response. In contrast, the 45° laminate exhibited matrix- and interface-dominated fracture involving shear deformation, interfacial debonding, and local delamination. In this case, post-curing may have improved the fiber–matrix adhesion and stress transfer efficiency, thereby increasing the energy required for crack initiation and propagation despite the slightly more brittle behavior of the epoxy matrix.

Failure analysis performed after tensile testing confirmed that fracture mechanisms strongly depend on laminate architecture. Laminates with fibers oriented at 90° were dominated by fiber breakage, whereas 45° laminates exhibited extensive matrix cracking and shear-dominated fracture. Hybrid laminates showed a combination of fiber fracture, matrix cracking, and interlaminar delamination. The relatively greater reduction in tensile strength observed for the hybrid [90°/45°/90°] laminate after prolonged SO_2_ exposure may be related to the presence of heterogeneous interfaces between adjacent layers with different fiber orientations. These interfaces are characterized by more complex stress distributions and may be more susceptible to local interlaminar stresses, moisture penetration, and interfacial debonding. As a result, degradation may preferentially initiate in the interlaminar region, leading to local delamination and a more pronounced loss of load-bearing capacity compared to laminates with a uniform fiber orientation. Additional testing of interlaminar shear strength would be required to confirm this assumption. These fracture mechanisms correspond well with previously reported failure modes of CFRP laminates under tensile loading, where damage typically develops through a combination of fiber fracture, matrix cracking, and interfacial debonding [42,43,44].

Overall, the obtained results confirm that proper selection of laminate architecture and thermal processing conditions plays a crucial role in optimizing the mechanical performance and environmental durability of CFRP composites used in engineering applications. The trends observed in the present study are in good agreement with previously published results on CFRP laminates, particularly regarding the beneficial effect of post-curing, the lower tensile performance of off-axis laminates, and the progressive degradation caused by humid and corrosive environments.

However, additional analyses such as DMA or DSC would be required to distinguish between the effects of increased cross-linking and residual stress relaxation and to quantify the degree of cure and glass transition temperature. Additional characterization using Fourier transform infrared spectroscopy would be required to determine whether the observed degradation is dominated by reversible plasticization or by irreversible chemical scission within the epoxy matrix and interfacial region.

## 5. Conclusions

This study evaluated the effects of fiber orientation, thermal post-curing, and corrosive SO_2_ environment on tensile strength and impact toughness of three-layer CFRP laminates manufactured from carbon fabric and epoxy resin. Based on the results obtained, the following conclusions can be drawn:

1. Fiber orientation was the dominant factor affecting the mechanical response of the investigated laminates. In the reference condition, the 90° laminate exhibited the highest tensile strength (484 MPa), whereas the 45° laminate showed the lowest value (93 MPa). The hybrid [90°/45°/90°] laminate provided intermediate tensile strength (330 MPa) and the highest impact toughness (16.8 J·cm^−2^).
2. Post-curing at 80 °C for 10 h improved the mechanical performance of all laminate configurations. Tensile strength increased by approximately 14% for the 90° laminate, 42% for the 45° laminate, and 59% for the hybrid laminate. The increase may be associated with an increased degree of cross-linking and improved fiber–matrix adhesion. These effects are consistent with the more compact fracture morphology and reduced fiber pull-out observed after post-curing.
3. Exposure to the humid SO_2_ environment caused progressive deterioration of the matrix and fiber–matrix interface, which is consistent with the observed SEM fracture morphology. After 14 exposure cycles, the tensile strength of the 90° laminate decreased from 484 MPa to 213 MPa, while the hybrid laminate retained a more balanced combination of strength and impact resistance.
4. Failure analysis confirmed that fracture mechanisms strongly depended on laminate architecture. The 90° laminates were dominated by fiber breakage, the 45° laminates by matrix cracking and shear failure, whereas the hybrid [90°/45°/90°] laminate showed combined fracture mechanisms and provided the most favorable balance between mechanical performance and environmental durability.

From an application perspective, hybrid laminate [90°/45°/90°] combined with post-curing appears to provide the most balanced combination of tensile strength, impact toughness, and environmental resistance. Similar trends in mechanical performance of CFRP laminates after post-curing and environmental exposure have been reported in previous studies dealing with durability of polymer matrix composites [45,46].

From a practical point of view, the obtained results indicate that hybrid CFRP laminates combined with thermal post-curing are suitable for structural applications requiring a balanced combination of tensile strength, impact resistance, and durability in aggressive industrial or transportation environments containing humidity and sulfur-based pollutants.

Future research should also include a wider range of fiber orientations and stacking sequences, as well as other aggressive environments, such as salt spray, alkaline solutions, acidic media, and cyclic temperature–humidity exposure, in order to provide a more comprehensive assessment of the long-term durability of CFRP laminates. Additional characterization using DSC, DMA, and FTIR would also be beneficial to quantify the degree of cure, glass transition temperature, and chemical degradation mechanisms in the epoxy– matrix and at the fiber–matrix interface.

## Figures and Tables

**Table 1:** Raw materials used for preparation of CFRP laminates and their basic properties.

Material	Basic Parameters
Carbon fiber fabric	Carbon woven fabric; areal weight: 160 g·m^−2^; plain weave; nominal thickness: 0.20–0.25 mm; density: 1.75–1.80 g·cm^−3^; tensile strength of carbon fibers: approximately 3500 MPa; elastic modulus: approximately 230 GPa.
Epoxy resin L285	Low-viscosity laminating epoxy resin; density at 25 °C: 1.18 g·cm^−3^; viscosity at 25 °C: 600–900 mPa·s; tensile strength: approximately 70–80 MPa; elastic modulus: approximately 3.0 GPa; glass transition temperature after post-curing: approximately 80–90 °C.
Hardener H286	Amine curing agent for epoxy resin L285; density at 25 °C: 0.95 g·cm^−3^; viscosity at 25 °C: 50–100 mPa·s; recommended mixing ratio with L285: 100:40 by weight.
Peel ply Interglas 98685	Polyamide peel ply; areal weight: 93 g·m^−2^; used for excess resin removal and surface preparation.
Breather fabric	Polyester breather fabric; areal weight: 200 g·m^−2^; used to ensure vacuum distribution and absorption of excess resin.
Release wax FRP HL-603	Mold release agent used to facilitate demolding of the cured laminate.

Suppliner—Havel Composites CZ s.r.o., Svésedlice, Czech Republic.

## Data Availability

The original contributions presented in this study are included in the article. Further inquiries can be directed to the corresponding author.
